# Advances in bioleaching of waste lithium batteries under metal ion stress

**DOI:** 10.1186/s40643-023-00636-5

**Published:** 2023-03-10

**Authors:** Xu Zhang, Hongjie Shi, Ningjie Tan, Minglong Zhu, Wensong Tan, Damilola Daramola, Tingyue Gu

**Affiliations:** 1 State Key Laboratory of Bioreactor Engineering, East China University of Science and Technology, Shanghai, 200237 China; 2 Department of Chemical and Biomolecular Engineering, Institute for Sustainable Energy and the Environment, Ohio University, Athens, Ohio 45701 USA

**Keywords:** Bioleaching, Metal ion stress, Biofilm, Waste lithium battery, Electrochemistry

## Abstract

In modern societies, the accumulation of vast amounts of waste Li-ion batteries (WLIBs) is a grave concern. Bioleaching has great potential for the economic recovery of valuable metals from various electronic wastes. It has been successfully applied in mining on commercial scales. Bioleaching of WLIBs can not only recover valuable metals but also prevent environmental pollution. Many acidophilic microorganisms (APM) have been used in bioleaching of natural ores and urban mines. However, the activities of the growth and metabolism of APM are seriously inhibited by the high concentrations of heavy metal ions released by the bio-solubilization process, which slows down bioleaching over time. Only when the response mechanism of APM to harsh conditions is well understood, effective strategies to address this critical operational hurdle can be obtained. In this review, a multi-scale approach is used to summarize studies on the characteristics of bioleaching processes under metal ion stress. The response mechanisms of bacteria, including the mRNA expression levels of intracellular genes related to heavy metal ion resistance, are also reviewed. Alleviation of metal ion stress via addition of chemicals, such as spermine and glutathione is discussed. Monitoring using electrochemical characteristics of APM biofilms under metal ion stress is explored. In conclusion, effective engineering strategies can be proposed based on a deep understanding of the response mechanisms of APM to metal ion stress, which have been used to improve bioleaching efficiency effectively in lab tests. It is very important to engineer new bioleaching strains with high resistance to metal ions using gene editing and synthetic biotechnology in the near future.

## Recycling technology for waste lithium batteries

In the past ten years, the lithium battery industry has developed rapidly around the world. Lithium batteries are mainly classified into two main categories, lithium metal batteries (LMBs) and lithium-ion batteries (LIBs). LIBs mainly include lithium iron phosphate batteries, lithium cobalt oxide batteries, lithium manganese batteries, and ternary lithium batteries. In recent years, LIBs have become the main power batteries because they can be charged repeatedly. With increasing consumer demand for portable electronics and electric vehicles, the production of LIBs is increasing rapidly (Zheng et al. 2018; Chen et al. 2019). However, since the lifespan of LIBs is usually less than 3 years in consumer electronics, and roughly 10 years in electronic vehicles (EVs), the amount of waste lithium-ion batteries (WLIBs) is increasing rapidly every year. According to published reports, the amount of WLIBs will increase from 10,700 tons in 2012 to 464,000 tons in 2025 and reach approximately 11 million tons by 2030 worldwide (Zhao et al. 2019; Lv et al. 2021). The natural resources for the production of LIBs are dwindling (Chen et al 2017). At the same time, toxic metals, such as lithium, nickel, cobalt, and manganese in WLIBs, as well as chemical compounds, such as electrolytes and binders, pose serious threats to the environment and human health if untreated. Therefore, legislations on the recycling of WLIBs have been implemented in many countries around the world (Reuter and Kojo 2012). The new EU (European Union) battery regulatory framework will increase materials recovery rate from 50 to 65% by 2025, and 70% by 2030 (Halleux 2021).

Hydrometallurgy and pyrometallurgy have long been used to recover metals from WLIBs (Chagnes and Pospiech 2013; Jha et al. 2013; Meshram et al. 2015; Pagnanelli et al. 2016; Pinna et al. 2017; Yang et al. 2014; Zeng et al. 2014). Although these traditional methods have high metal recovery rates, their processes pollute the environment. For example, pyrometallurgy can emit toxic furans and dioxins, and its process is energy intensive (Pagnanelli et al. 2016; Zeng et al. 2014; Xu et al. 2008). In traditional hydrometallurgy, strong acids, such as sulfuric acid, are used to dissolve metals in WLIBs. Thus, the generated acid-containing waste requires downstream treatment to meet waste discharge limits (Jha et al. 2013; Pagnanelli et al. 2016; Zeng et al. 2014; Hendrickson et al. 2015). Recently, a technology of combining organic acid produced by microorganisms with hydrogen peroxide has been tested. It may be a beneficial alternative to traditional hydrometallurgy (Ozairy et al. 2021).

**Fig. 1:**
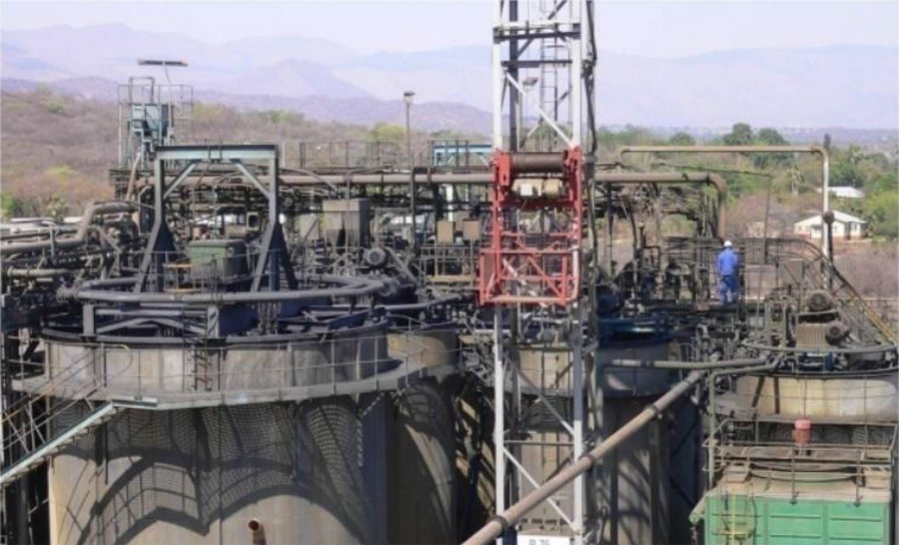
Commercial-scale BIOX® bioleaching process in Barberton, South Africa (Kaksonen et al. 2014). [Image courtesy of Dr. Anna H Kaksonen, The Commonwealth Scientific and Industrial Research Organization, Australia]

Nowadays, bioleaching is intensively investigated as a promising environmentally -friendly method to recover valuable metals from urban solid wastes rich in valuable metals, including waste circuit boards, WLIBs, coal fly ash, wastewater treatment plant sludge, steel slag, spent catalysts, and spent coin cells. (Gomes et al. 2018; Gu et al. 2018; Haragobinda et al. 2019; Hosseinzadeh et al. 2021). Bioleaching has been commercially practiced in copper mining (Petersen 2016) and it has been industrialized in refractory gold ore, copper ore, and uranium ore. Figure 1 shows such an example of commercial process. The world’s largest bioreactor is an open-pit copper mining facility (Carrasco et al. 2015). Up to now, more than 30% of the global copper production is produced through bioleaching technology, as well as 5% of gold and smaller amounts of cobalt, nickel, uranium, and zinc (Schippers et al. 2013; Brierley and Brierley 2013; Johnson 2013).

**Fig. 2:**
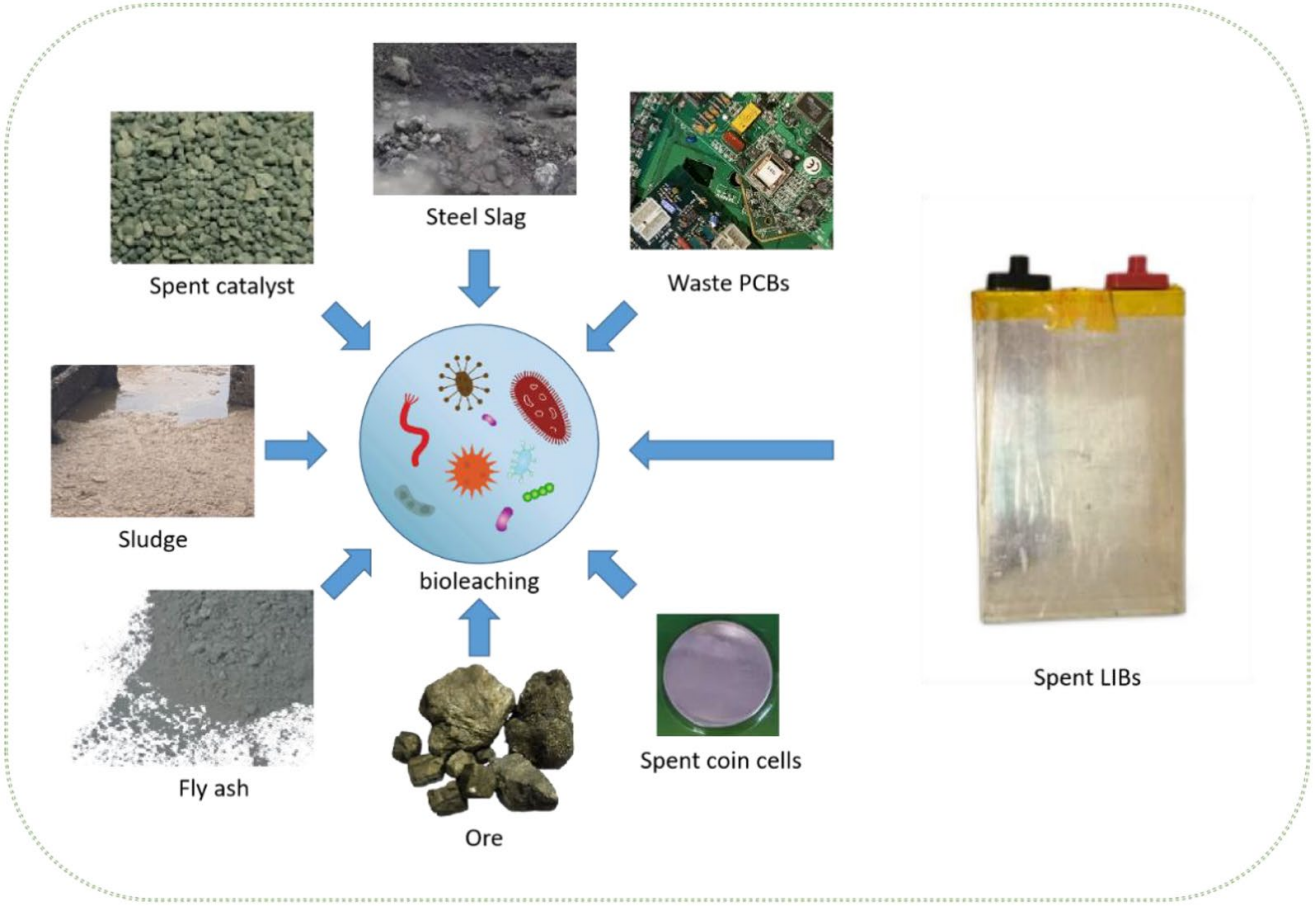
Examples of bioleaching applications

Bioleaching can effectively reduce carbon footprint, since carbon dioxide can serve as the carbon source for bioleaching chemoautotrophs (Banerjee et al. 2017). Therefore, bioleaching has the potential for industrialization in the sustainable recovery of valuable metals from urban mining (Zhou et al. 2019). Figure 2 shows various bioleaching applications to treat different wastes, including electronic wastes. It is interesting to note that space biomining is an exciting new development direction (Cockell et al. 2020).

The cathode materials of WLIBs often contain lithium alloy metal oxides; therefore, the cathode materials have become the main raw materials for bioleaching of WLIBs. During the bioleaching of WLIBs, microorganisms and their metabolites are used to convert metal elements in cathode material powders from insoluble state to soluble ions. Therefore, the leachate contains a mixture of various metal ions, such as Li^+^, Co^2+^, Ni^2+^, Mn^2+^, and Fe^3+^. In order to separate various metal components from the leachate, metal ions are precipitated by stepwise addition of alkali, oxalate, or carbonate (Gratz et al. 2014; Verma et al. 2019), or by electrodeposition (Zhang, et al. 2021). Finally, the regeneration of cathode materials can be accomplished by hydrothermal synthesis or calcination (Yang et al. 2017; Zhao et al. 2020).

**Fig. 3:**
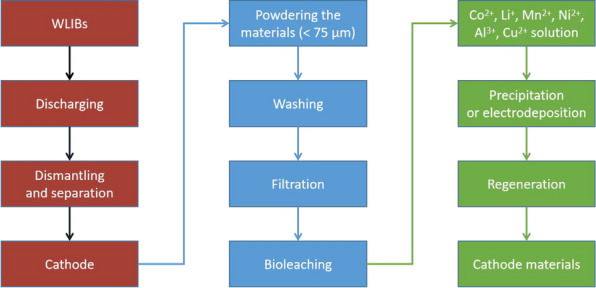
A process flowsheet for bioleaching WLIB

A proposed WLIBs bioleaching process flowsheet is illustrated in Fig. 3. In general, bioleaching is more simplified, economical, environmentally benign and less energy intensive than traditional hydrometallurgy and pyrometallurgy (Xin et al. 2009; Jegan Roy et al. 2021). It has a much longer cycle time, but its much larger scale can trade space for time, leading to a competitive total production output over a long time frame. However, the many engineering challenges in bioleaching, including slow kinetics, low pulp density (i.e., solid waste powder loading) (Moosakazemi et al. 2022), and low bacterial activity in addition to long leaching cycle (Pathak et al. 2017), seriously restrict the industrialization of bioleaching of WLIBs. Moreover, the activities of APM and the efficiency of the WLIBs bioleaching process will decrease over the time course of bioleaching in the challenging operating conditions, which mainly include three-phase gas–liquid–solid systems, low pH, high oxidation state, and high metal ion concentrations. These harsh bioleaching conditions have a series of adverse effects on the APM in a bioreactor, such as uneven material transfer and mixing, osmotic stress, acid stress, oxidative stress, and metal ion stress.

During the bioleaching process, metal ions will gradually accumulate in the leachate and therefore microorganisms must withstand these high metal ion concentrations for bioleaching to continue. It is found that high metal ion concentrations consistently limited the pulp concentration in bioleaching WLIBs (Liu et al. 2020a, b). This obviously becomes a key parameter impacting bioleaching efficiency. Therefore, this review focuses on the adverse impact of metal ions on the growth and metabolic activity of APM and bioleaching efficiency as well as strategies used to counter the impact. A multi-scale approach, including reactor scale, biofilm scale, cell scale, and gene scale, is used to address this critical bioleaching issue.

## WLIBs bioleaching technology and microorganisms

## Bioleaching process and mechanisms

Traditional engineering methods to improve the efficiency of bioleaching processes include strain screening, mutagenesis to create more potent strains, strain domestication and transformation, development of various bioleaching methods (one-step method, two-step method, and spent-medium method), optimization of process conditions (temperature, pH, pulp density, energy source, and aeration rate), and medium optimization. An optimized bioleaching process has a significant cost advantage over other processes. Traditional methods treat the interactions between microorganisms and solid particles as a black box due to a lack of understanding of the underlining mechanisms. It is necessary to reveal the response mechanism of bioleaching microorganisms to harsh environments, such as metal ion stress, and their regulatory strategies to improve the bioleaching efficiency.

**Fig. 4:**
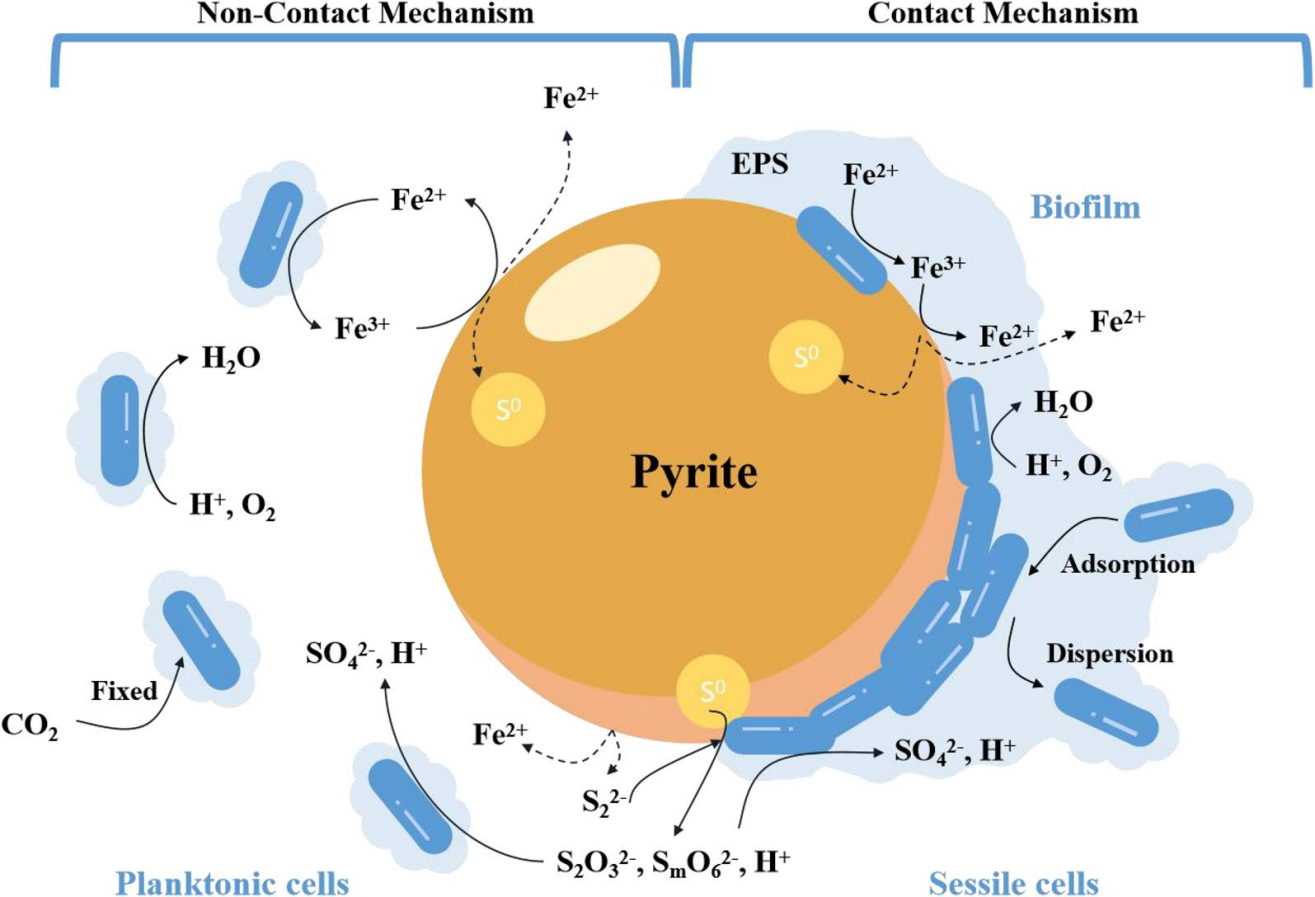
Bioleaching performance by contact and non-contact microbial cell

Bioleaching mechanisms can be described on the basis of microorganism–ore interactions: either contact leaching mechanisms or non-contact leaching mechanisms (Crundwell 2003; Boxall et al. 2018; Srichandan et al. 2020) as shown in Fig. 4. The contact leaching mechanism refers to the adsorption of APM on the surface of sulfide ore (energy source) through its own hydrophobicity or electrostatic attraction, and the formation of biofilms. These microorganisms in biofilms are called sessile cells. The sessile cells of APM continuously oxidize sulfide ores under aerobic conditions through iron oxidation and sulfur oxidation and finally transfer the electrons generated by sulfide ores to oxygen molecules. The specific reactions in this kind of contact leaching are shown below (Hansford and Vargas 2001):

1 $${\text{MS }} + {\text{ 2H}}^{ + } + \, 0.{\text{5O}}_{{2}} \to {\text{M}}^{{{2} + }} + {\text{S}}^{0} + {\text{ H}}_{{2}} {\text{O,}}$$

2 $${\text{S}}^{0} + {\text{ 2H}}^{ + } + {\text{ 2O}}_{{2}} \to {\text{H}}_{{2}} {\text{SO}}_{{4}} ,$$

in which MS represents the metal element M in a sulfide ore.

The non-contact leaching mechanism means that the APM cells do not directly attach to the surface of the sulfide ore. These microbial cells suspended in a liquid phase are called planktonic cells. The ferrous ion in the solution is used as the energy substance for the planktonic cells of APM. It is oxidized to ferric ion. The ferric ion oxidizes and decomposes the sulfide ore, and then releases ferrous ion and generates elemental sulfur, thereby providing energy for APM. In this cycle, APM can continuously oxidize and decompose sulfide minerals for growth and metabolism. The specific reactions of the non-contact leaching mechanism are shown below (Mishra et al. 2005):

3 $${\text{2Fe}}^{{{2} + }} + {\text{2H}}^{ + } + \, 0.{\text{5O}}_{{2}} \to {\text{2Fe}}^{{{3} + }} + {\text{ H}}_{{2}} {\text{O,}}$$

4 $${\text{MS }} + {\text{ 2Fe}}^{{{3} + }} \to {\text{2Fe}}^{{{2} + }} + {\text{ M}}^{{{2} + }} + {\text{ S}}^{0} .$$

When both leaching contact and non-contact leaching mechanisms are present, it is called a combined action of both contact and non-contact mechanisms (Hansford and Vargas 2001).

It is reported that the capabilities of sessile cells on a solid surface to produce acid and to resist harsh environment are stronger than that of planktonic cells, which is closely related to the biofilm formation with synergistic microbes and quorum sensing (Diza et al. 2018; Flemming and Wingender 2010), in addition to locally high volumetric cell mass. APM can produce locally high acidity at the bottom of microbial biofilm on solid particles. Moreover, APM will form biofilms with completely different morphological structures and functions under Li^+^ and Co^2+^ stress (Liu et al. 2022). It has been observed that the contribution of biofilm to copper extraction rate was as high as 50.5% in one study (Yang et al. 2014). Therefore, it is necessary to investigate the impact of metal ions on APM biofilms.

## Diversity of bioleaching microorganisms for WLIBs

Based on the nutrients provided, the bioleaching microorganisms can be divided into heterotrophic microorganisms (mainly heterotrophic bacteria and fungi) and chemoautotrophic microorganisms (mainly chemoautotrophic bacteria and archaea). Although their leaching mechanisms and methods are different, these microorganisms can secrete inorganic acids, organic acids, oxidizing substances, or cyanide to leach the valuable metals from metals-rich waste solids (Baniasadi et al. 2019). For example, heterotrophic fungi can promote the dissolution of valuable metal oxides and sulfides through the metabolic syntheses of citric acid, malic acid, acetic acid, succinic acid, and other organic acids (Rasoulnia and Mousavi 2016). One group of researchers used *Aspergillus niger* (a filamentous fungus) to study the bioleaching of WLIBs. Their results showed that the leaching efficiency of organic acids, such as citric acid, secreted by *A. niger* was higher than that of chemical leaching (Horeh et al. 2016). The chemical reactions between an organic acid and lithium cobalt oxide, a cathode material for lithium cobalt oxide batteries, are shown below:

5 $${\text{4LiCoO}}_{{2}} + {\text{ 12C}}_{{4}} {\text{H}}_{{6}} {\text{O}}_{{5}} \to {\text{4LiC}}_{{4}} {\text{H}}_{{5}} {\text{O}}_{{5}} + {\text{4Co}}\left( {{\text{C}}_{{4}} {\text{H}}_{{5}} {\text{O}}_{{5}} } \right)_{{2}} + {\text{ 6H}}_{{2}} {\text{O }} + {\text{ O}}_{{2}} ,$$

6 $${\text{2LiCoO}}_{{2}} + {\text{ 7H}}_{{2}} {\text{C}}_{{2}} {\text{O}}_{{4}} \to {\text{2LiHC}}_{{2}} {\text{O}}_{{4}} + {\text{ 2Co}}\left( {{\text{HC}}_{{2}} {\text{O}}_{{4}} } \right)_{{2}} + {\text{ 4H}}_{{2}} {\text{O }} + {\text{ 2CO}}_{{2}} ,$$

7 $${\text{2LiCoO}}_{{2}} + {\text{ 4H}}_{{2}} {\text{C}}_{{2}} {\text{O}}_{{4}} \to {\text{Li}}_{{2}} {\text{C}}_{{2}} {\text{O}}_{{4}} + {\text{ 2CoC}}_{{2}} {\text{O}}_{{4}} + {\text{4H}}_{{2}} {\text{O }} + {\text{ 2CO}}_{{2}} .$$

In addition to secreting organic acids, fungi can also secrete cyano compounds to complex metal ions. *Chromobacterium violaceum* uses intracellular glycine as substrate to form cyanide ion (CN^−^) and HCN through oxidative decarboxylation reaction. Cyanide can complex with heavy metal ions to form water-soluble metal cyanides (Faramarzi et al 2004). The reactions below explain this process using gold as the metal to be dissolved:

8 $${\text{NH}}_{{2}} {\text{CH}}_{{2}} {\text{COOH }} + {\text{ O}}_{{2}} \to {\text{HCN }} + {\text{ CO}}_{{2}} + {\text{ 2H}}_{{2}} {\text{O,}}$$

9 $${\text{4Au }} + {\text{ 8CN}}^{ - } + {\text{ O}}_{{2}} + {\text{ 2H}}_{{2}} {\text{O}} \to {\text{4Au}}\left( {{\text{CN}}} \right)^{{{2} - }} + {\text{ 4OH}}^{ - } .$$

Acidophilic iron–sulfur-oxidizing bacteria are the most widely used chemoautotrophic bacteria in the commercial bioleaching process. They can obtain energy by oxidizing low valence iron and sulfur in ores without the requirement for organic carbon nutrient. They mainly include the following genera in bioleaching: *Acidithiobacillus*, *Sulfobacillus*, and *Leptospirillum* (Norris et al. 2000; Marcináková et al. 2016). The chemical reactions between acidophilic iron–sulfur-oxidizing bacteria and lithium cobalt oxide are shown below. Furthermore, studies have shown that the reduced proteins in extracellular polymers substances (EPS) can effectively improve the bioleaching efficiency of cobalt (III) from lithium cobaltate (Wu 2020). Moreover, the study found that the domestication of a microbial consortium by using lithium-ion battery materials can improve the species abundance of the microbial consortium and increase the bioleaching efficiency of waste lithium-ion batteries (Cai et al. 2021).

10 $${\text{2S}}^{0} + {\text{3O}}_{{2}} + {\text{2H}}_{{2}} {\text{O}} \to {\text{4H}}^{ + } + {\text{ 2SO}}_{{4}}^{{{2} - }} \left( {\text{bacterial metabolism}} \right),$$

11 $${\text{Fe}}^{{{2} + }} - {\text{ e}}^{ - } \to {\text{Fe}}^{{{3} + }} \left( {\text{bacterial metabolism}} \right),$$

12 $${\text{4LiCoO}}_{{2}} + {\text{ 12H}}^{ + } \to {\text{4Li}}^{ + } + {\text{ 4Co}}^{{{2} + }} + {\text{ 6H}}_{{2}} {\text{O }} + {\text{ O}}_{{2}} ,$$

13 $${\text{Fe}}^{{{2} + }} + {\text{ LiCoO}}_{{2}} + {\text{ 4H}}^{ + } \to {\text{Fe}}^{{{3} + }} + {\text{ Co}}^{{{2} + }} + {\text{ Li}}^{ + } + {\text{ 2H}}_{{2}} {\text{O,}}$$

14 $${\text{2FeSO}}_{{4}} + {\text{ 2LiCoO}}_{{2}} + {\text{ 4H}}_{{2}} {\text{SO}}_{{4}} \to {\text{Fe}}_{{2}} \left( {{\text{SO}}_{{4}} } \right)_{{3}} + {\text{ 2CoSO}}_{{4}} + {\text{ Li}}_{{2}} {\text{SO}}_{{4}} + {\text{ 4H}}_{{2}} {\text{O}}{.}$$

Generally speaking, the optimum pH of chemoautotrophic bacteria is within the range of 1–3 in which metal ions can exist stably. Such bacteria also have a high tolerance to heavy metal ions as a result of long-term evolution (Quatrini and Johnson 2019). Most of the known strains and consortia reported recently in bioleaching of WLIBs are summarized in Table 1.

**Table 1:** A summary of recent bioleaching studies on the recovery of valuable metals from WLIBs

Microbe	LIB waste type	Recovery yield	Initial pH	Culture medium	Spent LIBs Pulp Density	Temperature	Method	Refs.
Single species bacterium								
*A. nige*r (PTCC 5010)	cathode and anode powder	Li 100%, Co 38%, Cu 94%, Mn 72%, Ni 45%	2.5	Sucrose medium	1.0 (w/v)	30 °C	One-step bioleaching	Bahaloo-Horeh et al. (2018)
*A. niger* (PTCC 5210)	Cathodes and anodes powder	Co 64%, Ni 54%	5.44	Sucrose medium	1% (w/v)	30 °C	Spent-medium bioleaching	Bahaloo-Horeh et al. (2017)
*A. niger*	Cathodes and anodes powder	Li 100%, Cu 100%, Mn 77%, Al 75%	5.44	Sucrose medium	2% (w/v)	30 °C	Spent-medium bioleaching	Bahaloo-Horeh et al. (2017)
*A. niger* (isolated)	Spent Li-ion Battery Powder	Li 100% Co 82%	2.4	Sucrose medium	0.25% (w/v)	30 °C	One-step bioleaching	Biswal et al. (2018)
*A. niger* (PTCC 5210)	Cathodes and anodes powder	Li 95%, Co 45%, Cu 100%, Mn 70%, Ni 38%	6	Sucrose medium	1.0 (w/v)	30 °C	Spent-medium bioleaching	Horeh et al. (2016)
*Penicillium citrinum*	Spent coin cells (SCCs) powder	Mn 53%, Li 70%	6	3.9% (w/v) PDA medium	20 g/L	30 °C	One-step bioleaching	Naseri et al., (2022)
*A. ferrooxidans* (ATCC19859)	Cathode waste materials of LIBs	0.5% Li 9%, Co 65%	2.5	9 k medium + S power + Fe^2+^ ion 3 g/L	0.5% (w/v)	30 °C	One-step bioleaching	Mishra et al (2008)
*A.s thiooxidans* (PTCC1717)	Anodes and cathodes powder	Li 99%, Co 60%, Ni 20% (3% w/v)	2.0	9 k Medium + S powder 5 g/L	1.0–5.0% (w/v)	30 °C	two-step bioleaching	Naseri et al.,(2019b)
*A. ferrooxidans* (PTCC1647)	Anodes and cathodes powder	Li 100%, Co 88%, Mn 20% (4% S/L)	2.0	9 K medium + FeSO_4_∙7 H_2_O 44.2 g/L	1.0–10% (w/v)	30 °C	Two-step bioleaching	Naseri et al., (2019a)
*A. thiooxidans*	spent coin cells powder	Li 99%	2	9 K medium + FeSO_4_∙7H2O (44.2 g/L)	30 (g/L)	30 °C	Two-step bioleaching	Naseri et al. (2019a)
*A. thiooxidans* (80,191)	Spent Li-ion Battery Powder	Li 22%, Co 66%	3.3 and 2.4	Basel 317 + S power 1%	0.25% (w/v)	30 °C	One-step bioleaching	Biswal et al. (2018)
*A. ferrooxidans* (DSMZ 1927)	Electrodes powder	Co 82% Li 89%, Mn 92%, Ni 90%	2.0	Modified 9 K medium + FeSO_4_∙7H_2_O 150 g/L	10% (w/v)	30 °C	Two-step bioleaching	Roy et al (2021a, b)
*A. ferrooxidans* (isolated)	LiNixCoyMn1-x-yO2	Li 31%,Mn 42%,Co 23%, Ni 23%	1.0	Single basic medium + S powder 16 g/L + pyrite 16 g/L	1.0% (w/v)	30 °C	One-step bioleaching	Xin et al., (2016)
*A. thiooxidans* (isolated)	LiNixCoyMn1-x-yO2	Li 85%,Mn 19%,Co 10%, Ni 10%	1.0	Basic medium + S powder 16 g/L + pyrite 16 g/L	1.0% (w/v)	30 °C	One-step bioleaching	Xin et al., (2016)
*A. ferrooxidans*	LiCoO_2_ powder	99.9% Co after 6 days	2.0	Modified 9 K medium + FeSO_4_∙7H_2_O 44.8 g/L + Copper 0.75 g/L	1% (w/v)	35 °C	One-step bioleaching	Zeng et al. (2012)
*A. ferrooxidans*	Cathode material powder	98.4% Co	2.0	9 K medium + FeSO_4_∙7H_2_O 444.8 g/L	1% (w/v)	35 °C	Two-step bioleaching	Zeng et al. (2013)
Bacterial consortia								
*A. thiooxidans, L. ferriphilum and A. ferrooxidans*	cathodes LiNi_x_CoyMn_1-x-y_O_2_ powder	Li 100%, Ni 42%, Co 40%, Mn 40%	1.0	A mineral salt medium with sulfur (1.0% w/v) + FeSO_4_∙7H_2_O 20 g/L	4% (w/v)	30 °C	Two-step bioleaching	Wang et al. (2022a, b, c)
*A. ferrooxidans* and *A. thiooxidans*	Cathode powder from laptop LIB	Li 60%, Co 53.2%, Ni 48.7%, Mn 81.8%, Cu 74,4%	1.8	Basal salts medium (pH 1.8) with both110 g/L soluble FeSO_4_·7H_2_O and 5 g/L sterile S powder	10% (w/v)	22 °C	Spent-medium bioleaching	Boxall et al. (2018a)
Locally isolated bacterial strains	cathode (LiCoO2) powder	Li 62.83%	7	3 g/l of meat extract, 5 g/l of peptone and 5 g/l of NaCl	2 (mg/mL)	30 °C	One-step bioleaching	Hartono et al.(2017)
*A. ferrooxidans* (PTCC1647) and *A. thiooxidan*s (PTCC1717)	Electrodes	Li 99.2%, Co 50.4%, Ni 89.4%	1.5	Modified 9 k medium + S powder 5 g/L + FeSO_4_ ∙7H_2_O 36.7 g/L	4.0% (w/v)	32 °C	Two-step Bioleaching	Heydarian et al (2018)
Acidophilic Microbial Consortia	LiCoO_2_ powder	Li 98.1%, Co 96.3%	1.25		5% (w/v)	42 °C	Two-step bioleaching	Liu et al. (2020b, a)
*A. ferrooxidans* and *A. thiooxidans*	Cathode powder	Li 80%, Co 67%	1.5	K_2_HPO_4_ 0.1 g/L, (NH_4_)_2_SO_4_ 2.0 g/L, KCl 0.1 g/L, MgSO_4_∙7H_2_O 4.0 g/L, FeSO_4_∙7H_2_O 44.2 g/L, S powder 4 g/l	1% (w/v)	30 °C	One-step bioleaching	Marcincakova et al. (2016)
*Alicyclobacillus* spp. and *Sulfobacillus* spp.	Electrodes powder	89% Li, 72% Co	1.0	Basic medium + S powder 16 g/L + pyrite 16 g/L	2% (w/v)	35 °C	Two-step bioleaching	Niu et al., (2014)
Mixed culture of sulfur-oxidizing and iron-oxidizing bacteria	Electrodes powder	Co 90%, Li 80%	1.0	9 K medium + S + FeS_2_	1% (w/v)	30 °C	Two-step bioleaching	Xin et al. (2009)
*A. ferrooxidans* (isolated) and *A. thiooxidans* (isolated)	LiNixCoyMn1-x-yO2, LiMn2O4 and LiFePO4	Li 98%, Ni 97%, Co 96%, Mn 90%	1.5	Basic medium + S powder 16 g/L + pyrite 16 g/L (1:1 ratio)	1.0% (w/v)	30 °C	One-step bioleaching	Xin et al. (2016)
*A. caldus and Sulfobacillus thermosulfidooxidans*	spent LiCoO_2_ batteries powders	99% of Co and 100% of Li	2.5	Modified 9 K + S powder 10 g/L + 6 g/L Fe^2+^	20 g/L	30 °C	Two-step bioleaching	Liao et al. (2022)

## Metal ion stress response in bioleaching microorganisms for WLIBs

### Toxicity of heavy metal ions toward bioleaching microorganisms

With the bioleaching process of natural ores and urban mines, a large amount of metal ions will be dissolved gradually and accumulated in the leachate (Zhao et al. 2008; Zhu et al. 2003). It is known that a trace amount of metal ions is very important for the growth and metabolic activity of microorganisms because they serve as enzyme co-factors (Alshiyabet al. 2008). However, when the concentrations of metal ions are high, they become cytotoxic. The current research in the literature on the direct toxicity of metal ions to APM mainly focuses on Ag^+^, Li^+^, Co^2+^, Ni^2+^, Hg^2+^, Bi^3+^, and Cu^2+^ (Huang et al. 2020; Liu et al. 2020a, b; Wu et al. 2018).

The cytotoxic mechanism of metal ions to APM is mainly manifested in the following five aspects. (1) Metal ions can replace the essential metals at the protein binding sites. (2) The sediment produced by metal ions entering the cell will destroy the integrity of cell membrane, damage the normal physiological function of organelles, and finally induce apoptosis (Bruins et al. 2000). (3) Metal ions can cause abnormal synthesis of intracellular biological macromolecules and affect the transcription and translation of genetic information (Bruins et al. 2000). For example, copper ions can change the conformation of proteins by covalently modifying the cysteine and histidine groups of proteins, leading to their inability to perform normal biological functions (Fan et al. 2004). The effects of metal ions on cellular genetic information are mainly manifested in interfering with the repair of intracellular DNA. It is found that cobalt and nickel can cause a DNA double strand to break during DNA replication, slowing down the replication process, and inhibit the biological function of RecBCD, which is a key enzyme in SOS repair pathway (Kumar et al. 2017). (4) Metal ions can cause metabolic disorders and affect electron transfer kinetics. For example, nickel ions can prolong the lag phase of *Thiobacillus ferrooxidans*, affect the oxidation rate of Fe^2+^, and lead to cell death (Li 2001). Numerous reports have shown that APM can obtain energy through the biological oxidation process of pyrite, which is closely related to the detoxification mechanism of heavy metals by microorganisms (Bruins et al. 2000). (5) Metal ions can cause reactive oxygen species (ROS) damage (Jones et al. 2013; Kong and Lin 2010;  Stadtman 2006). Under the metal ions stress, hydroxyl radicals formed by Fenton reaction and Haber Weiss reaction can often be detected, which can cause oxidative stress in microorganisms. For example, Co^2+^ can inhibit the activity of the enzymes GSH-Px (glutamine peroxidase) and CAT (catalase) related to the revenge of excess intracellular ROS in APM, resulting in the accumulation of intracellular ROS and cell oxidative damage and ultimately reducing the bioleaching efficiency of WLIBs (Wu et al. 2020).

Therefore, the key to improving the efficiency of bioleaching is to enhance the resistance of bacteria to heavy metal ions, so that a bioleaching process can proceed to a greater extent without premature shutdown.

### Detoxification mechanism of microorganisms against heavy metal ions

**Fig. 5:**
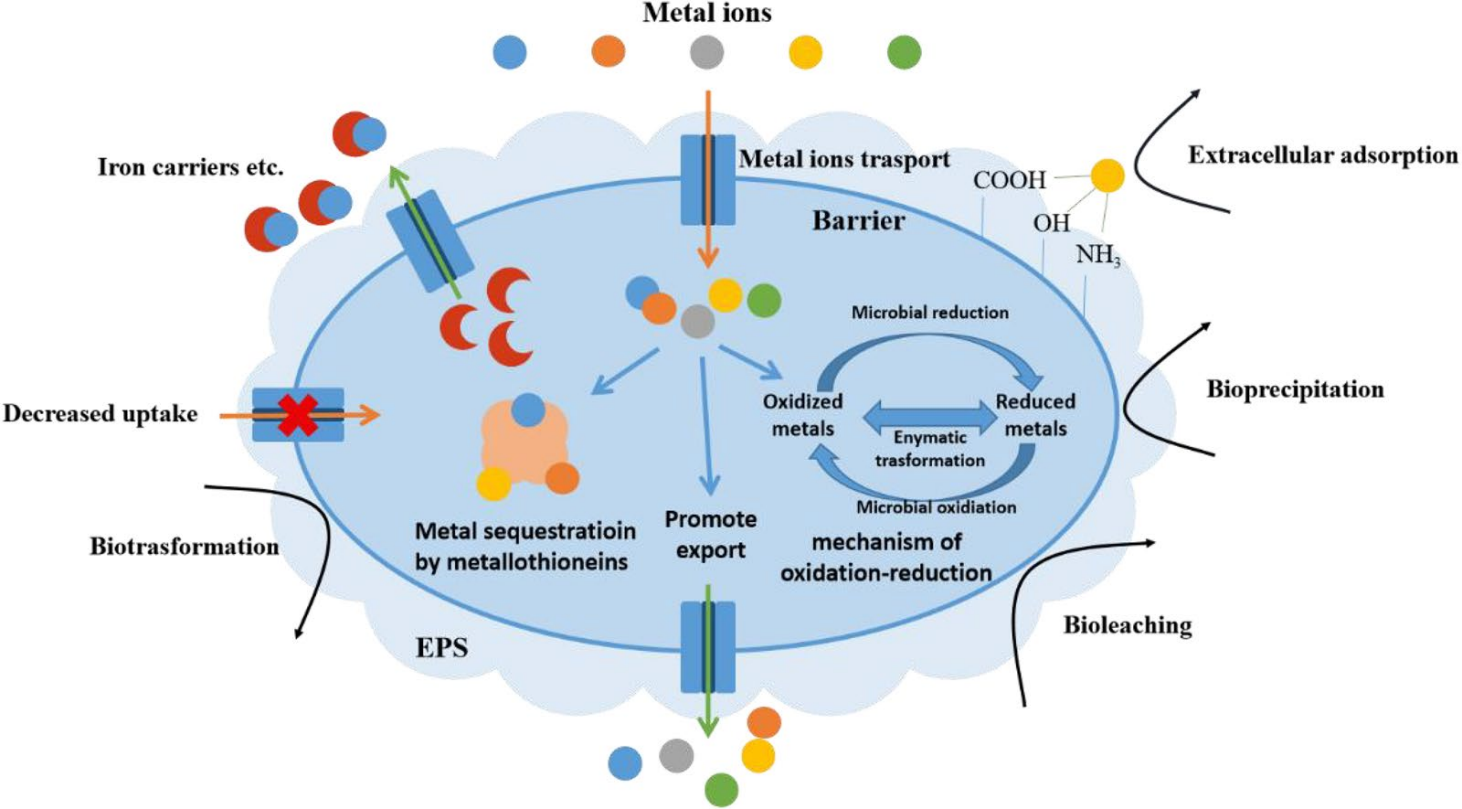
Various detoxification mechanisms for microorganisms under metal

Due to long-term natural evolution, bioleaching microorganisms have a certain degree of resistance to metal ions. They can reduce or eliminate the negative effects of heavy metal ions by self-regulating their physiological characteristics and maintaining their activities of growth and metabolism under metal ions stress. It is found that the detoxification mechanism of microorganisms to metal ions can be divided into extracellular isolation mechanism and intracellular detoxification mechanism as shown in Fig. 5.

#### Extracellular isolation mechanism

The extracellular isolation of heavy metal ions can be divided into cell wall barrier, adsorption of EPS and extracellular chelate precipitation. It can also change the permeability of the cell membrane to restrict the entry and exit of metal ions. Studies have found that microbial EPS can complex metal ions through carboxyl, hydroxyl, amino, amide group, and other functional groups, reduce the toxic effect on cells, and further prevent environmental metal ions from entering the cell, which is a protective barrier of bacteria against the toxicity of extracellular metal ions (Horn et al. 2001; Liu et al. 2022). For example, EPS produced by *Sulfobacillus thermosulfidooxidans* have strong adsorption properties for copper, nickel, zinc, and cadmium. The deprotonation of carboxyl and phosphoryl groups in EPS also contributes to the adsorption of heavy metal ions (Huang et al. 2020).

As a class of small molecular weight compounds secreted by most bacteria, iron carriers can chelate with metal ions, which is a common resistance mechanism to metal ions in most bacteria (Dimkpa et al. 2009). In addition to iron carriers, different microorganisms can also secrete other small molecular compounds to chelate heavy metal ions (Mathivanan et al. 2021).

Generally speaking, extracellular isolation mechanism can bind metal ions to the outside of bacterial cells and block metal ions from entering cells, thus reducing the toxicity of metal ions to the microorganisms.

#### Intracellular detoxification mechanism

Although heavy metal ions can be effectively isolated outside the cell through extracellular isolation mechanism, some metal ions will inevitably enter the cell through the transport channels on the cell membrane and affect the normal physiological activities (Joho et al. 1995). Microbial regulation of intracellular metal ions mainly includes the intracellular chelation of metal ions, converting metal ions into low toxicity or even non-toxic forms, and transporter efflux of metal ions. (Dopson et al. 2003; Vera et al. 2003).

Metallothionein is a low molecular protein rich in cysteine and mercaptan in microbial cells. It can chelate with heavy metal ions through sulfhydryl groups, so that microorganisms can maintain normal growth and metabolism in an environment rich in metal ions. Some studies have shown that the expression of bacterial metallothionein will be increased, and the overexpression of metallothionein also increases the proportion of heavy metal ions chelated under high concentrations of metal ions (Mathivanan et al. 2021; Dar et al. 2013).

The biotransformation of heavy metal ions by bacteria is mainly realized through physiological metabolic processes, such as oxidation–reduction and methylation. For example, the resistance mechanism of copper tolerant bacteria screened from a Brazilian copper mine was studied and found that the bacteria reduced free Cu^2+^ to non-toxic elemental Cu^0^ (Gracioso et al. 2021).

Transporters on the cytoplasmic membrane can respond to the changes in the environment, thus regulating the concentrations of heavy metal ions in cells through the energy provided by ATP hydrolysis, which is the most common heavy metal adaptive regulation mechanism of bacteria. Czca protein is a member of the resistance-nodule-cell division (RND) protein family and it can significantly improve the resistance of *Ralstonia metallidurans* to cobalt, zinc, cadmium, and nickel. As another member of the RND protein family, CBA transporter is closely related to bacterial resistance to cobalt and nickel. The cnrCBA efflux system consisting of structural genes *cnrC*, *cnrB,* and *cnrA* can pump Ni^2+^ out of the cell. P-type ATPase in *Mycobacterium* participates in Cu^2+^ transport, forms ion channels, and maintains intracellular Cu^2+^ homeostasis (Brown et al. 1995). On the other hand, as a transporter family driven by pH gradient and chemical concentration gradient, cation diffusion facilitators (CDF) protein family has different affinities for various metal ions. Under Ni^2+^ stress, *A. thiooxidans* in the co-culture system of *A. thiooxidans* and *L. ferriphilum* prefer to choose a more efficient Ni^2+^ transport pathway, which ensures that cells can expel heavy metal ions out of cells faster and more effectively (Xu et al. 2013).

Additionally, it is found that oxidative stress induced by metal ions can also damage cells. Studies have found that excessive intracellular ROS can lead to plasma membrane peroxidation, DNA damage, changes in enzyme activity, and apoptosis. The intracellular antioxidant system of microorganisms is mainly composed of two parts: enzymatic antioxidant system and non-enzymatic antioxidant system. These antioxidant systems reductively remove excess intracellular ROS and maintain a stable intracellular redox balance (Ray et al. 2012; Xu et al. 2016). However, when the concentrations of metal ions are too high, the intracellular antioxidant system cannot maintain the intracellular redox balance, resulting in the accumulation of ROS and cell damage. When glutathione and some other chemicals are added into the culture medium, ROS can be effectively removed, so that the bioleaching activity of microorganisms can be partially restored (Liu et al. 2020a, b).

As the most important detoxification mechanism of microorganisms to counter heavy metal ions, intracellular detoxification is relatively complex, and the differences among different microorganisms are obvious, which need to be further studied. Therefore, it is very important for a commercial bioleaching process to obtain natural or engineered bioleaching strains with strong metal ion tolerance. For example, it is found that the activities of microbial consortia biofilm commonly used in bioleaching are generally higher than that of single species (Gumulya et al. 2018). Thus, using microbiome methods to construct metallurgical microbial biofilm consortia with strong tolerance to metal ion toxicity is one of the future research directions (Gu et al. 2018).

## Biofilm of acidophilic microorganisms in bioleaching WLIBs

### Introduction to biofilm

It is shown that the long-distance interaction and adhesion between bacteria and mineral surface are very important for the formation of biofilm in bioleaching systems (Hall-Stoodley et al. 2004; Liu 2020). The adhesion of bacteria starts and strengthens the bioleaching process, which is closely related to the leaching efficiency (Li et al. 2019; Li et al. 2017; Diza et al. 2018; Zhu et al. 2003). For example, the efficiency of the bioleaching of chalcopyrite can be improved by directly strengthening the adhesion of *A. ferrooxidans* (Feng et al. 2020).

When microbial cells attach to the surfaces of solid particles, they will secrete EPS, which include polysaccharides, proteins, extracellular DNA, fibrin, lipids, and complex metal ions (Govender and Gericke 2011). EPS will embed the bacteria to form a three-dimensional polymerization network, namely, biofilm (Besemer et al. 2007). Biofilm is a micro-ecological environment that can provide an enclosure and protection for microbial community (Flemming and Wingender 2010; Flemming et al. 2007). Thus, most microorganisms in bioleaching systems live in a biofilm (Schippers et al. 2013; Castro et al. 2016). Compared with planktonic cells, the biofilm provides a much higher volumetric biomass density and a much more acidic local pH, and the synergy between various strains in a biofilm consortium makes it able to tolerate and survive in harsh bioleaching environments better (Ruiz et al. 2008; Vera et al. 2003; Hall-Stoodley et al. 2004; Jasu et al. 2021).

In an APM biofilm, the sessile bacteria have higher metabolic activities, and the volumetric density of sessile biomass can be 5–6 orders of magnitude higher than that of planktonic cells (Santegoeds et al. 1998). It has been reported that an APM biofilm can play a very important role in a WLIBs bioleaching process (Liu 2020). Therefore, it is necessary to study the key influencing factors to obtain efficient biofilms for WLIBs bioleaching process.

### Key factors influencing biofilm during bioleaching of WLIBs

Biofilm is a micro-ecological environment in which materials, energy, and information could be exchanged among the different microbial species to meet their needs of growth and metabolism. The key factors influencing biofilm formation are production of EPS, intercellular communication, metal ions, and motion-related function (Bellenberg et al. 2014; Besemer et al. 2007; Ruiz et al. 2008; Wolska et al. 2016; Wang et al. 2020a).

#### EPS

The formation of biofilm mainly includes adhesion, proliferation, and maturation stages (Abdallah et al. 2014), and the EPS secreted by microorganism are essential for biofilm formation (Fulaz et al. 2019). EPS are a layered liquid polymer with certain fluidity, which is mainly composed of extracellular polysaccharides, proteins, and nucleic acids (Govender and Gericke 2011; Naseri and Mousavi 2022). The amount of EPS secreted by the sessile bacteria growing on the surface of sulfide ore is high. It is mainly a viscous medium with certain fluidity composed of tight-type EPS and loose-type EPS. The former can be closely combined with the cell wall, and the latter can exchange materials and information with the external environment (Sheng et al. 2010). EPS are also the substrate for the microbial community in the biofilm to produce diversified spatial structures, to mediate the adhesion of bacteria in the biofilm to the contact surface of solid particles, and EPS can also be used as a barrier against some harsh environmental factors, such as osmotic pressure, antibiotics, and heavy metal ions (Diao et al. 2014). Thus, intercellular interactions may occur, including intercellular communication, horizontal gene transfer, and cooperative microflora formation. In addition, EPS can play the roles of an external digestive system by keeping extracellular enzymes close to microbial cells to metabolize dissolved colloids and solid biopolymers (Flemming and Wingender 2010).

It has been demonstrated that EPS can also play a very important role in interactions between bacteria, as well as that between bacteria and pyrite surface in a biofilm (Flemming and Wingender 2010). For example, it was found that furanone C-30 was used to inhibit the generation of EPS of *A. ferrooxidans* on the surface of ores and the expression of genes related to the formation of biofilm was down-regulated. Finally, the dissolution and bioleaching efficiencies of nickel and copper are inhibited (Zhao et al. 2015). Similarly, prior research shows that the amount of APM cells adhering to pyrite surface, the synthesis and secretion of EPS, and the structure of biofilm are closely related to the bioleaching efficiency of WLIBs (Liu et al. 2020a, b). For example, under the condition of Li^+^ and Co^2+^ stress, there is a decrease of EPS produced by APM, leading to a looser biofilm and a decrease in the bioleaching efficiency. Conversely, after the exogenous addition of spermine, the stress was relieved such that both the amount of EPS and the bioleaching efficiency of Li^+^ and Co^2+^ increased and the biofilms became denser (Liu et al. 2022). This indicates that there is indeed a close relationship between EPS in biofilms and WLIBs bioleaching efficiency, and its regulation mechanism needs to be further studied.

#### Signal molecule

Quorum sensing (QS) system is an important mechanism that controls the biofilm formation of bacterial attachment onto the surface of sulfide minerals. The signal molecules of QS are very important chemicals in biofilm formation and development. They enable bacteria to respond to the changes in the external environment and make adjustments in time (Gao et al. 2020; Hu et al. 2022). After initially adhering to the surface of pyrite during bioleaching process, APM will produce more endogenous acycloguanosine acid (Ross et al. 1985) by secreting a class of signal molecules, such as N-acyl homoserine lactone (AHL) (Ruiz et al. 2008) and endogenous cyclic dimer guanosine monophosphate (c-di-GMP) (Ross et al. 1985). Then, more planktonic bacteria are summoned by these signal molecules and attached to the pyrite surface, and subsequently they secrete a large amount of EPS to form a dense biofilm (Wolska et al. 2016). It is reported that the formation of *A. ferrooxidans* biofilm is enhanced by exogenous addition of long carbon chain AHL (González et al. 2013). On the other hand, it was found that furanone C-30 can inhibit the production of AHL, resulting in a looser *A. ferrooxidans* biofilm (Wenbin et al. 2011). Metal ions can affect the QS system by substituting the intracellular enzyme-binding site and forming a complex with AHL, thereby inhibiting biofilm formation that relies QS (Lami et al. 2022). Interestingly, it was found that cells in *Halobacterium sp.* biofilms from different locations adopted a particular shape, suggesting a functional differentiation. This demonstrated that even in the biofilm formed by a pure culture, there will be division of labor and cooperation through the QS system (Di Meglio et al. 2014).

The c-di-GMP compound, which widely exists in Gram-negative bacteria, is generally considered as the second messenger of QS, which can transform planktonic cells into sessile cells and promote the formation of biofilm (Römling et al. 2008). The intracellular c-di-GMP of *A. thiooxidans* is involved in the formation of Pel extracellular polysaccharide in the biofilm. The content of c-di-GMP in sessile bacteria is several times higher than that in planktonic bacteria (Díaz et al.^ 2018^).

It has been found that intercellular information transmission in biofilm is very important in mediating bacterial response to the changes in environmental conditions (Nadell et al. 2016). Therefore, an in-depth study on the information transmission mechanism of QS in APM biofilm will be helpful to understand the biofilm formation mechanism and to design corresponding regulatory strategies under metal ions stress.

#### Metal ions

Biofilm not only plays an important role in improving bioleaching efficiency, but also as an important mechanism for microorganisms to resist metal ions (Jeremic et al. 2016). A certain amount of metal ions can promote the formation of biofilm and maintain its thickness (Dey and Paul 2018). In particular, it often happens in the initial stages of the bioleaching process. For example, low concentration of Mg^2+^ can promote biofilm formation (Bellenberg et al. 2015). However, under the condition of Mg^2+^ stress, the formation of *A. ferrooxidans* biofilm is reduced due to the inhibition of the expression level of type IV pili-related genes (*PilA*, *PilW*, *PilV*, etc.). The bacterial activities are also inhibited by the interference of Mg^2+^ with carbon fixation metabolic pathways (Tang et al. 2018). Moreover, high (Mg^2+^) can reduce the formation of type IV pili and the adhesion of *A. ferrooxidans*, inhibit biofilm formation, and decrease the leaching rate of metal ions. Furthermore, the addition of acylated homoserine lactones (AHLs) can improve the resistance of *A. ferrooxidans* to Mg^2+^ by promoting biofilm formation (Tang et al. 2018). The single metal ions, such as Ag^+^, Hg^2+^, Mg^2+^, and Cu^2+^, can affect the activities of APM. Moreover, the study reported that under the influence of copper ions, it promoted the transfer of antibiotic resistance genes in the microbial community and increased the leaching efficiency of copper (Hu et al. 2022). There also exist some synergistic effects on microorganisms when different metal ions coexist. For example, the coexistence of Li^+^ and Co^2+^ can inhibit the activities of biofilm more seriously than a single ion species, leading to gradually reduced bioleaching efficiency of LiCoO_2_. It is also found that exogenous additives, such as glutathione (GSH) and spermine, can alleviate the damage of Li^+^ and Co^2+^ to the biofilm to a certain extent (Li and Ke 2000; Liu et al. 2020a, b; Liu et al. 2022). Therefore, the relationship between metal ion mixtures and biofilm formation needs to be further studied.

#### Motion-related function of APM

Biofilm formation is associated with microbial motility-related functions, such as chemotaxis and flagella formation. Chemotaxis is a physiological feature of the directional movement of cells under the guidance of chemical substances (Christel et al. 2018). Flagella are slender and wavy filaments attached to the bacterial body, which are mainly responsible for movement and a prerequisite for the settling of planktonic cells on a solid surface in the process of biofilm formation (Schuhmacher et al. 2015). It is found that under nickel ion stress, the chemotaxis of bacteria is severely inhibited, resulting in a loose and incomplete biofilm (Chen et al. 2022). Therefore, the relationship between motion-related functions of APM and biofilm formation needs to be further investigated.

In summary, the function of biofilm is crucial in the bioleaching of WLIBs, and therefore, it is necessary to study the structure and properties of biofilms. Simple and straightforward approaches capable of detecting structure and properties of biofilms should be developed. A better understanding of the structure–function relationship of biofilms during the process of bioleaching WLIBs is desired.

#### Microbial interaction

In addition to QS, microorganisms in biofilms also have synergy and cooperative relationships, such as cohesion and coaggregation, metabolic interaction, horizontal gene transfer, and production of beneficial components (Luo et al. 2021). At the stage of biofilm formation, the multiple microorganism species will form a mixed-culture biofilm by coaggregation on the surface of solid media (Zhang et al. 2015; Castro et al. 2016; Wang et al. 2020b). In the process of bioleaching a sulfide ore, the introduction of sulfur-oxidizing microorganisms into a pure culture or a mixed culture dominated by iron-oxidizing microorganisms is conducive to the oxidation of the passivation layer on the surface of the ore (Deng et al. 2017). Therefore, elemental S is oxidized to sulfuric acid, and the pH of the culture medium decreases, which inhibits the formation of the potassium ferrovanadium passivation layer (Gu et al. 2015) and ultimately improves the efficiency of bioleaching (Gu et al. 2013).

However, there are competition and antagonism among various microorganisms in a biofilm, such as competition for nutrients and production of deleterious components (Luo et al. 2021). During the initial stage of biofilm formation, there is competitive adsorption among different microorganisms. The adsorption of *Acidithiobacillus caldus* was greatly affected by the presence of *Leptospirillum ferriphilum* (Song et al. 2011). Some microorganisms will produce antagonistic substances to gain a growth advantage. Several *Sulfolobus* strains were found to produce protein toxins named Sulfolobicins, which can kill some microorganisms (Prangishvili et al. 2000). Some microorganisms will become dominant in a biofilm through the combined action of tunneling of swimmers and secretion of antimicrobial compounds (Di Meglio et al. 2014).

In conclusion, it is essential to study the symbiosis and antagonism among microorganisms in a biofilm. Moreover, the construction of a highly cooperative biofilm through artificial modulation will improve the stress resistance of microorganisms and bioleaching efficiency as a consequence.

### Methods for the detection and characterization of biofilms

At present, it has become a research hotspot to further understand the characteristics of the commonly used APM biofilm’s morphology using microscopic imaging techniques in bioleaching research. The combination of atomic force microscope (AFM) and epifluorescence microscope (EFM) by 4′6-diamidino-2-phenylindo (DAPI) staining and fluorescence in situ hybridization (FISH) probes demonstrated that sessile cells on pyrite coupons can be identified and assigned to a particular organism (Noël et al. 2010).

**Fig. 6:**
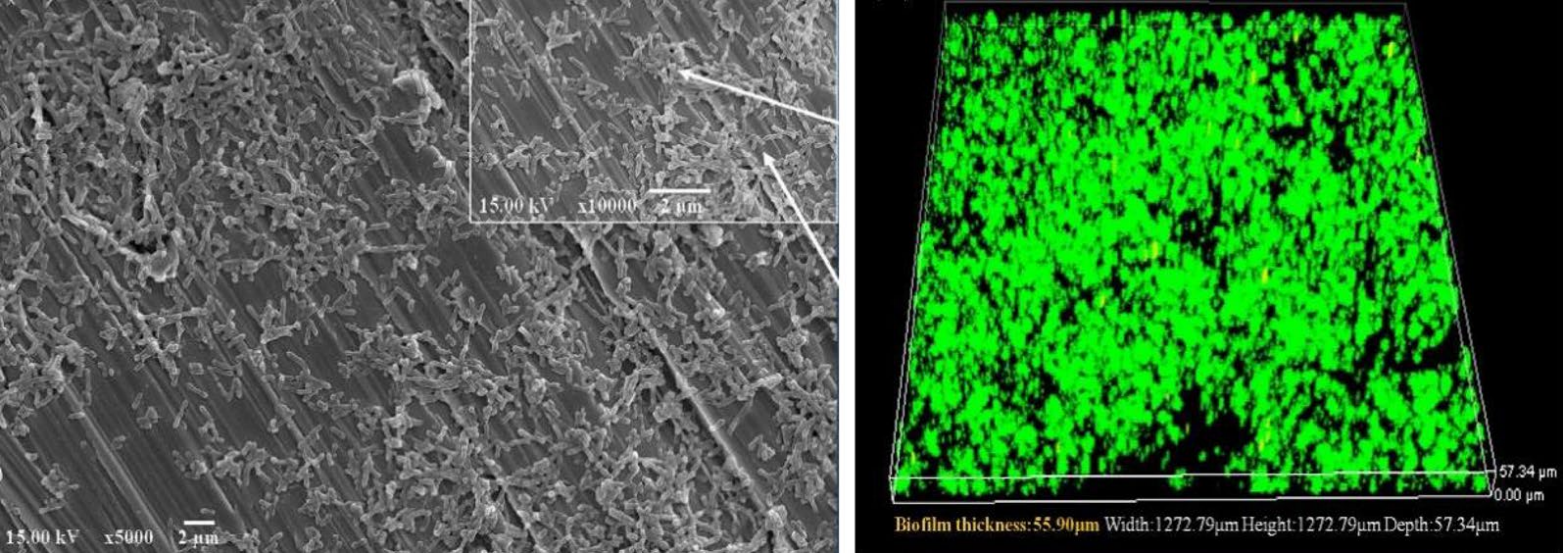
SEM (left) and CLSM (right) images of A. caldus SM-1 biofilm on S32654 SASS steel coupon surface [Dong et al. 2018]

Scanning electron microscopy (SEM) and confocal laser scanning microscopy (CLSM) have played important roles in studying the microstructure of biofilm. Some images are shown in Fig. 6. For example, it is observed that a large amount of bioleaching microorganisms accumulate on the surface of pyrite under SEM (Sanhueza et al. 1999). CLSM combined with different fluorescence labeled dyes can be used to conduct qualitative or quantitative research on the structure of biofilm, EPS composition, number of sessile bacteria, and dead/live cells ratio in the biofilm. It was found that (5Z)-4-bromo-5-(bromomethylene)-2(5H)-furanone (furanone C-30) can inhibit the formation of *A. ferrooxidans* biofilm using nucleic acid fluorescent dye SYTO 9 and EPS polysaccharide fluorescent dye Alexa Fluor 594 ConA in coordination with CLSM (Zhao et al. 2015). Fluorescent dyes SYTO 9 and propidium iodide (PI) are used to observe the bacterial biofilm structure under CLSM. The living and dead sessile bacterial cells can be observed and green and red dots under CLSM, and the amount of sessile bacteria can be estimated by their fluorescence intensity (Takenaka et al. 2001).

Biofilm activity is also important for bioleaching efficiency and studies have explored the correlation between these two characteristics. Researchers commonly use macroscopic parameters (free cell density, pH, and redox potential) instead of qualified parameters of biofilm to characterize the bioleaching kinetics. However, there are usually significant differences between these macroscopic and qualified parameters. For example, the volumetric cell density in the biofilm is often orders of magnitude higher more than that of the planktonic cells in the bulk liquid, and the pH value under the same biofilm can easily be 2 units lower than that in the bulk liquid (Vroom et al. 1999). This means that physical properties in the bulk fluid may be imprecise and misleading for the conditions underneath a biofilm above a solid surface.

APM can obtain energy by redox reactions in a biofilm. Therefore, extracellular electron transfer (EET) between a sulfide surface and a biofilm is directly related to their abilities to leach metal ions. A dense biofilm (i.e., more sessile cells) is beneficial to maintain the stronger EET rates. When sulfide ore is used as an electron source (energy source), it is corroded, because it is solid dissolution with electron loss. Parameters, such as EET rates and structure of microbial biofilm, have been characterized by electrochemical measurements of the corrosion by APM (Dong et al. 2018). Because the sulfide ore corrosion directly correlates to the electrons harvested by APM for energy production, the electrochemically measured corrosion rate is more precisely correlated with the health of the APM biofilm in bioleaching than the bulk-fluid pH which likely has a delayed response (Liu et al. 2022).

## Electrochemical monitoring of biofilm health in bioleaching

Modern sensors in many areas are mostly electrochemical. Electrochemical methods have been widely used in microbiologically influenced corrosion (MIC) investigations in the literature (Wang et al. 2022a). An electrochemical workstation equipped with linear polarization resistance (LPR), potentiodynamic polarization, and electrochemical impedance spectroscopy (EIS) software is used to measure the corrosion resistance, corrosion current density and corrosion impedance. Researchers found that S32654 SASS, a super austenitic stainless steel with excellent corrosion resistance commonly used in the construction of bioleaching reactors, was susceptible to MIC in an acidic environment (pH 3.3) caused by *A. caldus* SM-1 bioleaching bacterium. The electrochemical results showed that the pitting corrosion rate increased greatly in the presence of *A. caldus* SM-1 medium compared to the abiotic control at an initial pH of 3.3 because the biofilm damaged the stainless steel’s passive film in some spots (Dong et al. 2018).

Many MIC studies have verified that LPR, potentiodynamic polarization (PDP) and EIS scans show a good correlation with coupon weight loss and pitting corrosion data (Wang et al. 2022b). For corrosion caused by biofilm harvest of electrons, corrosion outcome depends on sessile cell counts because more sessile cells harvest more electrons (Jia et al. 2017a, b). Electron transfer details between a solid surface and a biofilm can be elucidated by equivalent circuit modeling of EIS spectra (Jia et al. 2017a, b; Poma et al. 2021).

Electrochemical methods have just started finding their ways in biofilm characterizations in bioleaching. APM can derive energy using pyrite ore during bioleaching, leading to the corrosion of pyrite. This corrosion process is similar to carbon steel corrosion by a sulfate reducing bacterium (SRB) biofilm, which can be monitored using the aforementioned electrochemical methods. A higher corrosion rate means that the APM biofilm has a higher metabolism activity level because more energy is harvested from the ore. Thus, it indicates a healthy biofilm not suffering from the toxicity of metal ion stress.

A classical electrochemical glass cell for corrosion testing typically consists of three electrodes: a working electrode (WE) to be corroded, a counter electrode, and a reference electrode. A lower open-circuit potential (OCP) for a (conductive) pyrite WE means that the WE has a higher tendency to lose electrons (to be corroded). When different ore mixtures are bioleached, the leaching efficiency of ores with lower OCPs was found much higher than that of ores with higher OCPs (Natarajan 1992). The electrochemical characteristics of pyrite and chalcopyrite were studied and results showed that the primary cell formed by pyrite and chalcopyrite reduced the ion exchange resistance and promoted the bioleaching efficiency of chalcopyrite (Zheng et al. 2021).

Bacteria can promote the oxidation (i.e., corrosion) of pyrite, which can be monitored using additional electrochemical methods such as potential polarization (Palencia et al. 1991). Cyclic voltammetry test results showed that bacteria damaged the passive film produced in the process of mineral leaching and thus accelerated the leaching of pyrite (Liu et al. 2008).

EIS has been used during the bioleaching of pyrite by *A. ferrooxidans*. The results showed that the inoculation of bacteria is beneficial to the oxidation of pyrite and can enhance the oxidation rates of the intermediate sulfur that formed in the mineral leaching process (Liu et al. 2011). It was reported that charge transfer resistance of *S. thermosulfidooxidans* increased significantly on the first day of bioleaching in the biological oxidation of minerals, which is related to the adhesion of bacteria on the mineral surface at the initial stage (Deng and Gu 2018). The EIS of *L. ferrooxidans* adhering to the chemically modified pyrite surface was measured (Saavedra et al. 2021). The results showed that when the bacterial biofilm adhered on pyrite surface, the low-frequency region of the EIS changed significantly, and the change of phase angle in the low-frequency region was directly related to the amount of sessile bacterial cells. The growth and metabolism of APM are closely related to the oxidation of pyrite, because this electron donor provides energy to APM.

Electrochemical frequency modulation (EFM) can be used for monitoring MIC (Beese et al. 2013; Wang et al. 2022b). Unlike EIS, which uses one AC (alternate current) signal, EFM uses two. This method has not been adopted to examine biofilms in bioleaching.

**Fig. 7:**
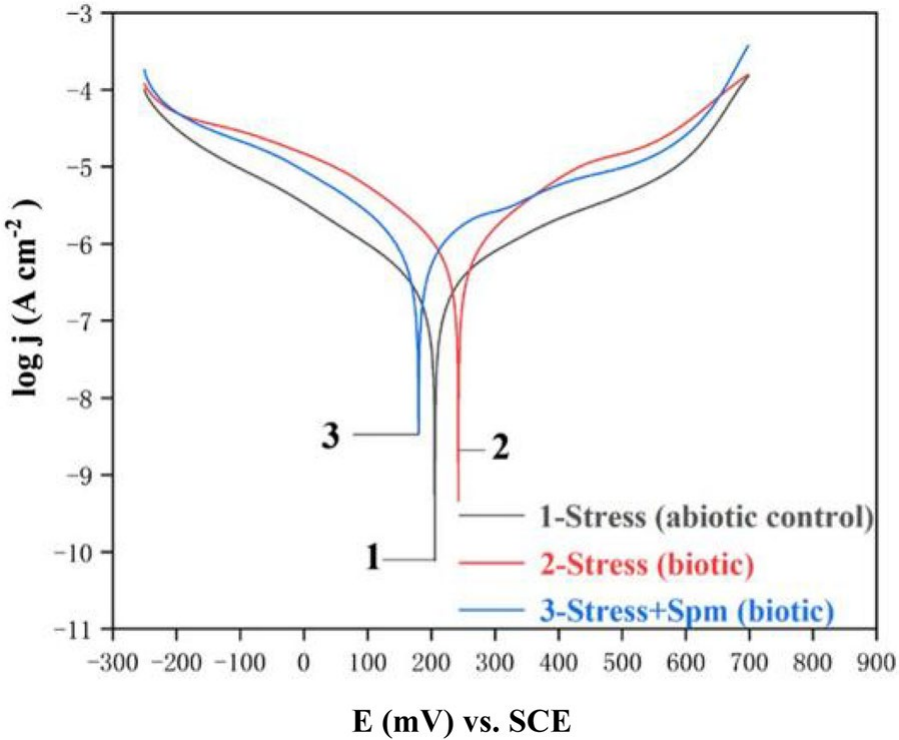
Tafel polarization curves of pyrite working electrode with and without biofilm coverage 7 days after Li^+^/Co2^+^ addition to simulate metal ion stress in bioleaching [Liu et al. 2022]

PDP scans, also known as Tafel scan, are widely used in corrosion monitoring to provide corrosion current density and pitting potential (Wang et al. 2022a). It was found that corrosion current density data from Tafel polarization curves can be used to monitor the ability of biofilm to obtain electrons from pyrite during acidophilic microbial consortium (AMC) bioleaching of Li_2_CoO_2_ (Liu et al. 2022). The AMC biofilm became somewhat loose under the stress of Li^+^ and Co^2+^. The corrosion potential (*E*_*corr*_) of pyrite covered with the AMC biofilm increased compared with the control without metal ion stress, suggesting that pyrite had a lower tendency to be corroded. More importantly, at the same time the corrosion current density (*j*_*corr*_) decreased, confirming that pyrite corrosion by the AMC biofilm decreased owing to the metal ion stress that inhibited the biofilm. When spermine is added, the biofilm became denser, the *E*_*corr*_ decreased and *j*_*corr*_ increased as shown in Fig. 7 and Table 2. These data suggest that Tafel analysis data for the pyrite WE reflected the biofilm health. A separate study showed that during the pyrite bio-oxidation process, *S. thermosulfidooxidans* biofilm gradually became denser. The results of Tafel analysis showed the *E*_*corr*_ in the biofilm gradually decreased, while the *j*_*corr*_ increased. In comparison, the biofilm became looser under nickel ion stress than the control, exhibiting lower corrosion. This suggests that the growth of *S. thermosulfidooxidans* biofilm was inhibited and the bio-oxidation process of pyrite was significantly affected by high concentration of Ni^2+^ (Chen 2022). It is consistent with the results that biofilms can promote EET, which is beneficial to the biological oxidation of pyrite in bioleaching (Gu et al. 2021).

**Table 2:** Tafel parameters from Tafel scans in Fig. 7 (data from [Liu et al. 2022])

System	*j*_corr_ (μA/cm^2^)	*E*_corr_ (mV) vs. SCE	*β*_a_ (mV/dec)	* − β*_c_ (mV/dec)
Abiotic control	0.453	205	281	245
Metal ion stress (biotic)	1.10	243	194	199
Metal ion stress + spermine (biotic)	2.00	180	509	264

Currently, there are two detection methods that can directly quantify the performance of bioleaching biofilms: pH monitoring and cell counting.

In pH monitoring, the acid-producing ability of biofilms can be detected by pH microprobes (Joshi et al. 2017). However, the pH microprobes are very fragile and difficult to handle. Due to their tiny size, they are very prone to poisoning by chemicals such as highly reactive sulfides. Monitoring bulk-fluid pH using a regular pH probe has the disadvantage of not getting the true pH underneath the APM biofilm, which can be much lower than the bulk-fluid pH (Vroom et al. 1999).

In cell counting, the cells in biofilm are first detached from the solid surface using mechanical or chemical means, and then counted using an assay kit, agar plating, or MPN (most probably number) serial dilution. These biofilm assay methods are not suitable for continuous online monitoring. Some methods, like plating and MPN, take days for incubation to finish.

Tafel scan provides near real-time corrosion rate. It can be scanned repeatedly if a proper scan scheme is followed (Wang et al. 2022a). If Tafel *j*_*corr*_ trends lower, it means a biofilm is harvesting less electrons. This can mean a weakening biofilm or biofilm vitality due to metal ion stress. This electrochemical method is more responsive than relying on bulk-fluid pH monitoring, which is indirect and is prone to delay.

In general, various electrochemical methods can be used for effective monitoring of the formation and development of APM biofilms during adhesion, growth, and death of microbial cells on the working electrode surface (Poma et al. 2021). If a pyrite ore is used in bioleaching, electrochemical monitoring can use a piece of pyrite cube or disk that is conductive with a surface of around 1 cm^2^ (standard for electrochemical tests) to serve as a WE (Liu et al. 2022). There are only a few bioleaching studies that deployed electrochemical corrosion monitoring tools. More research is needed to develop electrochemical sensors for online monitoring of biofilm health.

## Transcriptome analysis in bioleaching under the metal ion tress

Microorganisms can sense and respond to changes in many different environmental conditions, which is the basis for understanding the self-regulation mechanisms of bacteria in adapting to harsh environments. With the advances of omics, biofilm analysis nowadays can involve the use of transcriptomics, proteomics, and metabolomics to formulate effective strategies for studying the phenotypes of microorganisms in biofilms. The transcriptome technology has been used to comprehensively reveal the regulation mechanisms of bioleaching microorganisms from the gene expression level under metal ion stress (Feng et al. 2020; Zheng et al. 2015).

Since Selkov et al. (2000) published the genome sequence of *A. ferrooxidans* in 2000, more and more researchers have paid attention to bioleaching microorganisms using transcriptomics. There are 93 metallurgical microbial genomes publicly available in NCBI database, including 55 bacterial genomes and 36 archaeal genomes (Martinez et al. 2015). Therefore, transcriptomics is a powerful tool (more powerful than the real-time polymerase chain reaction tool) that is gaining popularity in explaining the metabolic regulation mechanisms at the gene level. It can provide insights for understanding the cellular response mechanisms of bioleaching microorganisms to metal ions.

### Metal ion transporter pathway

As an important detoxification method of bacteria, the intracellular transport pathway of metal ions is very important. It is found that the resistance to copper ions in *A. ferrooxidans* is closely related to the cation channel protein gene *afe0022* (Yuan 2010). The transcription level of the gene *afe0022* increases with the Cu^2+^ concentration. Under the stress of 0.4 mol/L Cu^2+^, the expression of the gene is up-regulated by 112 times.

It is indicated that under Mn^2+^, Zn^2+^, and Cd^2+^ stress, the expression levels of metal ion transporter protein genes *afe-0671*, *afe-0674*, *afe-143,* and *afe-1144* in *A. ferrooxidans* increase (Wu et al. 2013). The possible metal ion resistance determinants are identified in *A. ferrooxidans* in the presence of Cd^2+^. Changes in the expression levels of proteins involved in iron oxidation such as Cyc2 and Cyc1 and 50S ribosomal proteins L1 and L13 are observed. The expression levels of transporter protein genes (*lferr_0186*, *lferr_0209*, etc.) are significantly up-regulated (Javiera et al. 2019). In the late stage of chalcopyrite bioleaching, the expression levels of *cusA* and *czcA*, which are the transporter protein encoding genes in *S. thermosulfidooxidans,* are significantly up-regulated to resist the environmental stress caused by the accumulation of metal ions, toxins and metabolites (Ma et al. 2019). Therefore, APM can improve its own ability to resist metal ion stress through the expression of genes involved in metal ion transporter proteins.

### Oxidative phosphorylation pathway

Under the condition of metal ion stress, microbes will regulate the intracellular metabolic pathway and reduce the toxic and side effects of metal ions on the microorganism for self-protection. However, this process often requires microorganisms to consume more energy. Oxidative phosphorylation is an important energy-gaining process in prokaryotic microorganisms, which can supply electrons produced by oxidation of energy substances to ADP to synthesize ATP. Therefore, acidophilic iron–sulfur-oxidizing microorganisms can oxidize Fe^2+^ and sulfur in the environment through oxidative phosphorylation and synthesize ATP in cells (Quatrini et al. 2009; Bathe and Norris 2007). The transcriptome of *Morchella spongiola* under Cd^2+^ stress was analyzed with results showing that the oxidative phosphorylation pathway was very beneficial to the detoxification mechanism of Cd^2+^ (Xu et al. 2021). Researchers found that under the stress of Cu^2+^, the mRNA expression of F-type ATPase involved in oxidative phosphorylation pathway in *A. caldus* are significantly up-regulated, and the mRNA expression of NADH dehydrogenase is significantly down-regulated. Bacteria tend to produce more energy to resist extreme environmental conditions (Feng et al. 2020). Under Ni^2+^ stress, there are 11 up-regulated differentially expressed genes (DEGs) involved in the NADH-quinone oxidoreductase and cytochrome *c* oxidase pathways in *S. thermosulfidooxidans* resulting in its high resistance to Ni^2+^ (Chen et al. 2022). Therefore, APM can improve its own ability to resist metal ion toxicity through the expression of genes involved in the oxidative phosphorylation pathway to provide more energy.

### Two-component regulatory system

When the environment is rich in heavy metal ions, bacteria can recognize the changes of chemical signals in the external environment and regulate the bacteria to respond through the intracellular signal transduction system (Xu et al. 2013). The two-component system that commonly exists in bacteria can sense the changes of environmental signals through histidine protein kinases (HPK) on the cell membrane and transmits them to response regulators to regulate the processes of intracellular osmoregulation, bacterial chemotaxis, and metal ion induction. It was found that *Escherichia coli* relies on the transport system encoded by *nik* operon to recognize the Ni^2+^ concentration in the environment and regulate the intracellular Ni^2+^ concentration (Navarro et al. 1993). *Vibrio cholerae* can affect the expression of Ni^2+^ absorption system under the action of Fe^2+^ and heme, in which heme can bind to *nik* operon protein VCA1098 (Muranishi et al. 2022). The osmotic pressure in bioleaching can be significantly increased under metal ion stress, and the tolerance of bacteria to high osmotic pressure contributes to the improvement of bacterial growth activity (Wu 2020). A two-component system composed of KdpD and KdpE proteins was identified, which is ubiquitous in bacteria and archaea, can maintain the intracellular osmotic pressure of microorganisms by regulating the homeostasis of intracellular K^+^ (Ballal et al. 2007). Under Ni^2+^ stress, the expression of histidine kinase-related encoding gene *kdpD* and response regulator-related encoding gene *ddpE* in *S. thermosulfidooxidans* cells were significantly up-regulated. In addition, the expression of *cheA* and *cheY* genes involved in chemotaxis and flagella formation in the chemotactic family was significantly down-regulated. Finally, the formation of the bacterial biofilm was found inhibited (Chen et al. 2022).

Therefore, APM relies on a two-component system to sense the stimulation of the external harsh environment, and specifically turn on the self-regulation mechanism of two-component system to maintain the cytoplasmic stability and formation of biofilm and provide a suitable environment for intracellular enzymatic reaction. Thus, it is necessary to carry out more research using this two-component system.

### Genetic information processing pathway

The transmission of genetic information plays an important role in cell proliferation, material exchange, energy metabolism, and apoptosis. The higher concentrations of metal ions in cells can directly react with nucleic acid molecules and proteins, destroy the structure of signal recognition molecules, and eventually lead to the loss of normal physiological functions of biological macromolecules (Xiao et al. 2021; Kheirallah et al. 2021). Studies have shown that heavy metal ions can directly destroy the DNA damage repair pathway, combine with proteins in the DNA repair system, and block the signal transduction pathway of DNA damage repair (Kumar et al. 2017). Uranyl ions can be combined with the zinc finger structure of DNA repair protein PARP-1, replace the zinc ions in the zinc finger structure, and inhibit the activity of DNA repair protein (Zhou et al. 2021). It was found that Ni^2+^ can replace Fe^2+^ in the catalytic center of DNA repair enzyme ABH2 and destroy the active center of the protein (Chen et al. 2010). A group of researchers analyzed the resistance mechanism of microbial colonies in metal sediments by using metagenome sequencing technology (Yan et al. 2020). Their results showed that *proteus* reduced the DNA damage caused by metal ions through efficient DNA recombination and repair. Genes *ruvC* and *ssb* were up-regulated, which means that *S. thermosulfidooxidans* increased the expression of key enzymes in DNA replication and mismatch repair under Ni^2+^ stress, so as to ensure the normal expression and transmission of genetic information (Chen et al. 2022). It was found that *rcnA, czcD,* and *corC* genes are related to the expression of a Co resistance protein (Nanda et al. 2019). The *cadC* gene of transcriptional regulators in the ArsR/SmtB family can act as responsive transcriptional repressor for Co(II), Pb(II), Cd(II), and Zn(II) (Busenlehner et al. 2002). Additionally, *cusB* and *copA* are copper resistance genes (Orell et al. 2010), and the *aioA*, *arsC*, *acr3,* and *arsM* arsenic resistance genes (Nookongbut et al. 2017).

In order to ensure that genetic information can be accurately transmitted from the previous generation to the next generation, microorganisms must improve the activity of genetic information processing pathway to ensure the stability of genetic information (Kheirallah et al. 2021). For example, the APM in the bioleaching process relies on the genetic information processing pathway to ensure the normal intracellular DNA replication, transcription, and translation, guide the progress of intracellular biochemical reactions, and finally make the cells grow and reproduce normally. Therefore, it is necessary to analyze the genetic information processing pathway of APM in bioleaching process under metal ion stress.

### Glutathione metabolic pathway

It was found that glutamine can promote the scavenging of intracellular ROS in APM to a certain extent and thus significantly improve the bioleaching efficiency of Li^+^ and Co^2+^ by APM (Liu et al. 2020b, a; Inaba et al. 2021). Under Ni^2+^ stress, *idH*, *gltB,* and *cysE* genes in the glutathione metabolic pathway are significantly up-regulated in APM, and the scavenging ability of intracellular hydroxyl radical and superoxide anion is also enhanced. Thus, the ROS damage induced by a high concentration of Ni^2+^ is alleviated to a certain extent (Chen 2022). Therefore, it is necessary to study glutamine metabolic pathway of APM during the bioleaching process of WLIBs under metal ion stress.

**Fig. 8:**
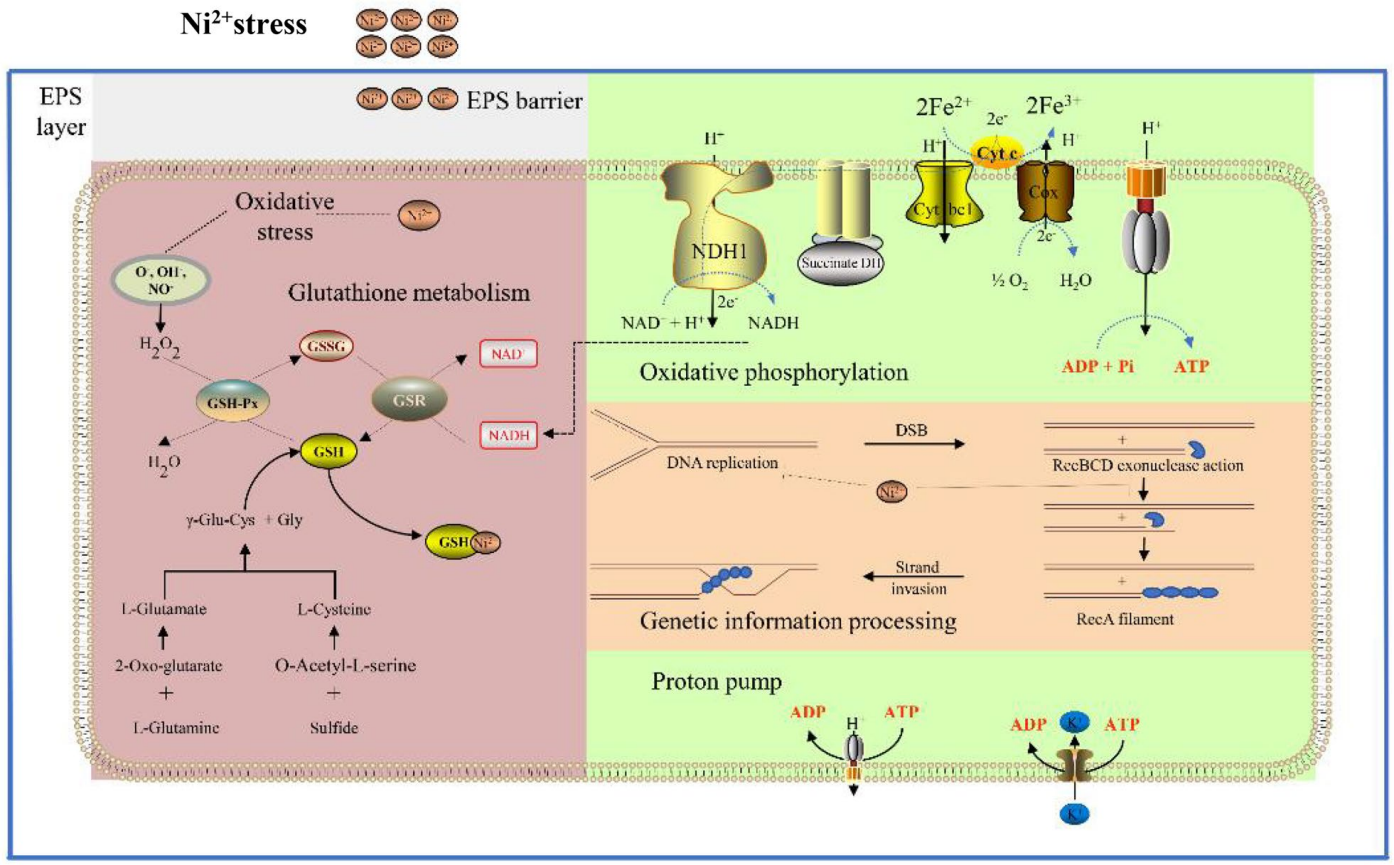
A putative mechanism model for *S. thermosulfidooxidans* adaptation to Ni2^+^ stress [Chen et al. 2022]

Microbial stress resistance is very important for APM to maintain normal physiological and biochemical performances under harsh environmental conditions. The study of microbial resistance genes can help understand the precise regulation mechanism for the microbial growth and metabolism under metal ion stress as illustrated in Fig. 8. A possible response mechanism of *S. thermosulfidooxidans* to Ni^2+^ stress can be investigated through a multi-scale approach. Under Ni^2+^ stress, the formation of a biofilm can be inhibited through the reduced mobility and QS regulation of *S. thermosulfidooxidans*. A large amount of EPS are produced as a protective barrier to isolate the bacteria from the extracellular Ni^2+^. At the same time, the extracellular osmotic pressure adjusting system, the ROS scavenging system, the ATP synthesis system, and the genetic information processing are activated to maintain the normal physiological state. A certain resistance of metal ion stress will be maintained according to the response measures above (Chen et al. 2022). At the same time, it is necessary to study the whole genome function of microorganisms and lay a foundation for gene editing and synthetic biology transformation in the construction of new strains (Gumulya et al. 2018).

Metallurgical microorganisms with higher resistance to extreme environmental conditions can be obtained by gene editing technology to improve the process efficiency of bioleaching electronic wastes (e-wastes) (Wiedenheft et al. 2012; Yamada et al. 2022). The CRISPR-Cas system is the most widely used gene editing tool (Ishino et al. 2018). Although the transformation of metallurgical microorganisms by gene editing technology is still in the development stage, current research is still in the aspects of exploring gene/genome functions and determining the key genes of cell phenotype and physiological functions (Shohei et al. 2022; Jung et al. 2022). It is believed that with the invention of new gene editing tools, researchers will be able to engineer desirable strains and process technologies in the near future. Ultimately, the goal of resource recycling of valuable metals in e-wastes will be achieved. Bacteria with special functions can be obtained based on gene editing methods. However, the genes related to the response to metal ion stress need to be clarified sufficiently to provide some guidance for identifying key editable genes.

## Summary and prospect

The stress of metal ions to metallurgical microorganisms can lead to the following problems in the bioleaching process of WLIBs: long leaching cycle, low pulp density, low bacterial activity, and low leaching efficiency. In order to speed up the industrialization of bioleaching, these problems need to be solved urgently. Therefore, it is necessary to understand the mechanisms of APM response to metal ion stress using a multi-scale approach. The typical approach may include investigating the microbial growth and bioleaching efficiency during bioleaching at reactor scale, the 3D morphology and electrochemical characteristics of APM biofilm at biofilm scale, the APM microbial resistance to metal ions at cell scale, and the expression of corresponding genes and genome of APM for metal ion resistance at gene scale. Based on these results, it will be helpful for the in-depth understanding of the response mechanisms of microorganisms in biofilms to metal ions.

The bioleaching efficiency of WLIBs can be improved by designing a novel bioreactor capable of in situ separation of heavy metal ions for continuous microbial cultivation tolerating a relatively high slurry concentration. The artificially domesticated microbes or direct construction of highly resistant microbial communities with the characteristics of overexpressed metal ion resistance genes, and EPS-related genes, can improve the microbial tolerance of heavy metal ions. Anti-oxidative stress, anti-osmosis stress, and QS signaling strategies can also be adopted to promote the formation of biofilms.

For monitoring biofilm health under metal ion stress during bioleaching. Specifically, this review endorses the use of electrochemical measurements (e.g., Tafel scan) for sensing the health of a biofilm, which is more responsive and direct than pH monitoring. Accordingly, regulatory strategies to improve biofilm resistance to metal ions will be developed. Furthermore, omics techniques, synthetic biology techniques and gene editing technology can be employed for the development of novel bioleaching microorganisms with high tolerance to metal ion stress, which will remove a key bottleneck in the industrialization of processes for bioleaching WLIBs.
